# Supplementary material for: Paratransgenic manipulation of a tsetse microRNA alters the physiological homeostasis of the fly’s midgut environment
Source: PLoS Pathog. 2021 Jun 9;17(6):e1009475. doi: 10.1371/journal.ppat.1009475 (PMC8216540; doi:10.1371/journal.ppat.1009475)
Supplement: S1 Table — (DOCX) [file ppat.1009475.s001.docx]

**S1 Table. qPCR primer list.**

| Target name | Gene ID* | Strand | Sequence | Tm (^o^C) |
| --- | --- | --- | --- | --- |
| *miR275* | GMOY012914 | F | TCAGGTACCTGAAGTAGCGC | 63.2 |
|  |  | R | GAATCGAGCACCAGTTACGC | 62.9 |
| *U6* | GMOY012690 | F | CGCAAGGATGACACGCAAA | 63.4 |
|  |  | R | GAATCGAGCACCAGTTACGC | 62.9 |
| *Gapdh* | GMOY000473 | F | CTGATTTCGTTGGTGATACT | 57.7 |
|  |  | R | CCAAATTCGTTGTCGTACCA | 60.6 |
| *Adgf*3 | GMOY012374 | F | TGGCATATGTTGGAGCAATT | 60.6 |
|  |  | R | TTCGATGATAGTCTGGTTTGC | 60.3 |
| *Adgf5* | GMOY012375 | F | GCGGCTTAAGAATGTTGAA | 58.6 |
|  |  | R | CATCCAGGAAAAGATCCCA | 59.0 |
| *SGP*1 | GMOY012268 | F | GAGGTGACGGAAAGAATGCC | 62.8 |
|  |  | R | CCTCCACCTCCAAGGAATC | 61.3 |

*[www.vectorbase.org](http://www.vectorbase.org)
