# Supplementary material for: Paratransgenic manipulation of a tsetse microRNA alters the physiological homeostasis of the fly’s midgut environment
Source: PLoS Pathog. 2021 Jun 9;17(6):e1009475. doi: 10.1371/journal.ppat.1009475 (PMC8216540; doi:10.1371/journal.ppat.1009475)
Supplement: S2 Table — (DOCX) [file ppat.1009475.s002.docx]

**S2 Table. Summary of reads mapping.**

| Treatment^a^ | Bio Rep^b^ | UMR to *Gmm*^c^ | Total reads^d^ | %^e^ |
| --- | --- | --- | --- | --- |
| Gmm^3xant-^*^miR275^* | PV1 | 93195093 | 99680737 | 93.5% |
|  | PV2 | 75181622 | 80568456 | 93.3% |
|  | PV3 | 109045602 | 118150796 | 92.3% |
| Gmm^Scr-275^ | PV1 | 80707959 | 87018339 | 92.7% |
|  | PV2 | 76359692 | 84419977 | 90.5% |
|  | PV3 | 70371662 | 76298372 | 92.2% |
| Gmm^3xant-^*^miR275^* | MG1 | 49165958 | 57907648 | 84.9% |
|  | MG2 | 50567526 | 59000219 | 85.7% |
|  | MG3 | 40358127 | 46427056 | 86.9% |
| Gmm^Scr-275^ | MG1 | 41904481 | 48496703 | 86.4% |
|  | MG2 | 43455822 | 51771676 | 83.9% |
|  | MG3 | 47341705 | 55365983 | 85.5% |

^a^Treatment: Gmm^3xant-^*^miR275^* = tsetse flies that were colonized with rec*Sodalis* expressing 3 times of antagomir275. Gmm^Scr-^*^275^* = tsetse flies that were colonized with rec*Sodalis* expressing scrambled *miR275* (as control).

^b^Bio Rep = biological replicate. PV = cardia; MG = midgut.

^c^UMR to *Gmm* = the number of reads that were uniquely mapped to *Gmm* transcript

^e^% = the percentage of UMR to *Gmm* transcript

^d^Total Reads = total number of raw reads that were mapped to *Glossina morsitans morsitans* (*Gmm*) transcript
