# Supplementary material for: Paratransgenic manipulation of a tsetse microRNA alters the physiological homeostasis of the fly’s midgut environment
Source: PLoS Pathog. 2021 Jun 9;17(6):e1009475. doi: 10.1371/journal.ppat.1009475 (PMC8216540; doi:10.1371/journal.ppat.1009475)
Supplement: Attachment — Submitted filename: Yang et al_response to reviewers.docx [file ppat.1009475.s006.docx]

3 May, 2021

Dear Editors and Reviewers,

We would like to thank you for your comprehensive review of our manuscript, entitled ‘Paratransgenic manipulation of tsetse microRNA alters the physiological homeostasis of the fly’s midgut environment’. In the following pages we provide an italicized response to each of the reviewer comments, which are listed in the order they originally appeared. Again, we thank the reviewers for taking the time to provide such valuable feedback about our research, and we hope the manuscript is now suitable for publication in PLoS Pathogens.

Sincerely,

Liu Yang, Brian L. Weiss, Adeline E. Williams, Emre Aksoy, Alessandra de Silva Orfano, Jae Hak Son, Yineng Wu, Aurelien Vigneron, Mehmet Karakus and Serap Aksoy

***Please note that the line numbers mentioned in the response to reviewers are for the revised manuscript (clean copy) that has no tracked changes, NOT for the version with tracked changes.***

**Part I - Summary**
Please use this section to discuss strengths/weaknesses of study, novelty/significance, general execution and scholarship.

Reviewer #1: The manuscript PPATHOGENS-D-21-00521 adds an important and exciting approach to down regulate gene expression in tsetse flies combining paratransgenesis and miRNA sponges. In addition, it adds an important contribution to understand the mechanisms used by Trypanosoma brucei parasite to manipulate its invertebrate host physiology, and further investigate the role of miR275 gene under this context.

Reviewer #2: In General, the research was well-performed, very well written, has very interesting results and the subject is of crucial importance to tsetse and the parasitic African trypanosomes control, which continues to be one the most prevalent infected disease in the world, in Africa. The authors made a complex and complete study about the manipulation of tsetse microRNA (miR275).
I have only some concerns. The authors could make the text clearer. The author summary is very well written but the abstract and introduction could be improved.
For example, on the first sentence of Abstract
“Tsetse flies are vectors of parasitic African trypanosomes (Trypanosoma spp.)” it is not necessary (Trypanosoma spp.), that could be deleted. They could also write in the abstract tsetse microRNA (miR275) instead of only "tsetse miR275 expression". In the introduction, the authors should make some modifications, adding more information about the Trypanosoma species, which is a complex genus.
Tsetse transmits two morphologically indistinguishable subspecies of the parasite that cause distinct disease patterns in humans, T. brucei gambiense and T. brucei rhodesiense. Why do not include this information? There are other Trypanosoma species very important, such as T. cruzi that causes the American trypanosomiasis, or Chagas diseases, transmitted by triatomines. Or Trypanosoma rangeli, that is also an American parasite transmitted by triatomines, more frequent than that by T. cruzi., but nonpathogenic to humans.
I suggest that the title could be changed to "Paratransgenic manipulation of a tsetse microRNA alters the physiological homeostasis of the fly’s midgut environment"

*We changed the title as suggested. We also added information about Trypanosoma species into the text.*

Reviewer #3: The manuscript is well written and presents a novel system for the use of transgenic bacterial symbionts to modify the expression of miRNAs in a disease vector. Previous studies have used insect symbiotic bacteria to produce dsRNA rather than miRNA antagomirs to affect gene expression, making the manuscript´s approach novel. The authors should refer to publications by Liu et al regarding the use of densovirus for a similar paratransgenic approach in Aedes albopictus. The study´s strength is the development of a novel paratransgenic system for antagomir expression and the application of omics to better understand the effects on midgut physiology of altering miRNA expression. The limitation is that there is no direct measurement of the amount of antagomirs produced by the bacteria in situ to get an estimate of the dose required for an effect. The limitation of the lack of antagomir quantification was addressed indirectly through the test of an in vitro model with insect cells to verify the efficacy of the effect. The suggestion that the amount of bacteria in the tissue can affect the level of inhibition of the miRNA needs to be verified, as the bacterial quantification and qPCR might need to be normalized for tissue weight for comparisons across tissues. For the quantification of the bacteria in midgut and cardia tissues, it might be more informative to normalize the bacterial count per mg of tissue to be able to compare between both tissues. The statistical approaches are appropriate.

*We added the reference as suggested.*

*We respectfully feel that our qPCR quantification of miR275 is sufficient in our study. We did not make a direct comparison of miR275 expression level between cardia and midgut, thus we feel it’s not necessary to normalize by tissue weight. Herein we normalized our miR275 level to the reference gene, which is the same way we performed in comparison of miR275 abundance between two tissues in a previous publication (which was cited in the discussion section). Our theory that the number of bacteria per tissue could affect the level of miRNA inhibition is one possible explanation for why we did not see significant miR275 knockdown in cardia.*

**Part II - No additional experiments required**

**Part III – Minor Issues: Editorial and Data Presentation Modifications**
Please use this section for editorial suggestions as well as relatively minor modifications of existing data that would enhance clarity.

**Reviewer #1:** I have few suggestions/comments to help to improve clarity and accuracy of the manuscript and listed them below:

Line 30: miR275 is mentioned here for the first time but with no background info. Readers would greatly benefit from a short description of its importance since it is the main molecular target of this study.

*As the reviewer suggested we added a brief description of miR275 in line 34.*

Line 31: Regarding the compromising effect on the PM integrity, I suggest adding the subsequent effect on facilitating the parasite establishment in the fly gut. Although lines 344 and 345 nicely reflects this correlation, readers would greatly benefit from having this info in the abstract section.

*As the reviewer suggested, we now describe the correlation between trypanosome infection and PM integrity in the abstract (line 29).*

Line 33: I suggest adding here the main hypothesis tested in this study.

*Although we do not state our hypothesis in the abstract, we do describe what we did in this study and how we did it.*

Line 37: Readers would benefit from a more informative info on "modulated infection outcomes".

*As the reviewer suggests, we changed this (in lines 37-39) to reflect precisely what we observed, which was an increase in fly survival following infection with a pathogen bacteria as well as an increase in trypanosome infection outcomes.*

Line 62: Are references #2 and 3# the best choice for the disease outcome if left untreated?

*We now provide more recent and relevant references related to this topic.*

Line 141: Why there are 5 nucleotides represented in black in the reverse 3xant-miR275 sequence? I would expect to find them matching with the 3-nucleotide linker sequence (atc) in the forward 3xant-miR275 sequence.

*We added an explanation (at now line 141), that includes a relevant reference, into the revised text. We indicate that the non-complementary sequences create a bulge at the linker sequence to increase effectiveness of the sponge construct.*

Lines 338 and 339: The percentages between control and experimental groups are switched.

*The control and experimental groups are not switched. 3xant-miR275 flies (experimental groups) have higher survival rates than the Scr flies (control groups)*.

Line 474 – Table S4: I suggest authors to add a new sub-sheet in Table S4 including these six genes. I could only find them in the DE sub-sheet.

*We have added a new sub-sheet as requested by the reviewer.*

Lines 518 to 521: To exclude the possibility of a systemic effect, authors should not rely on evaluating only three SG-specific genes expression in SG tissue. Other genes and tissues should not be ignored. Therefore, I must disagree with the section 3.7 subtitle (line 514) and conclusion (lines 527 to 529) stating that the effect observed is gut specific. Authors can only conclude that it does not have effect on these three SG-specific genes. The section 3.7 heading and its conclusion need careful rephrasing.

*We changed the 3.7 subtitle (line 508) to “Paratransgenic knockdown of miR275 is not observed in tsetse’s salivary glands”. We also rephrased lines 527-529 accordingly (now lines 521-523).*

Line 545: In this context, the mentioning of "be regulated" would require additional assays, therefore I suggest using "be influenced".

*We have made this change as the reviewer requested (line 539).*

Lines 577 and 578: Could authors provide references in the tsetse field that supports the statement “Increased midgut weight is indicative of impaired blood meal digestion and/or excretion”?

*The references (16 and 10) are listed at the end of the sentence (lines 571-573).*

Typos
Line 117: Add the Gmm abbreviation after "Glossina morsitans morsitans".
Line 273: Did authors mean "newly emerged" instead of "newly eclosed"?
Line 328: I suggest "physiological processes" instead of “physiologies”.
I did not review references format throughout the whole reference list, but found names with unusual capital letters (line 829), and italics format converted into text code (945 and 946).

*We have corrected all typos indicated by the reviewer.*

**Reviewer #2: (No Response)**

**Reviewer #3:** Abstract: "This paratransgenic system successfully knocked down miR275 levels in the fly’s midgut, which consequently obstructed blood digestion and modulated infection outcomes with an entomopathogenic bacteria and with trypanosomes."

It would be useful to include in the abstract by how much the expression was reduced and data on how digestion and infections were affected.

*We added the data into the abstract as suggested.*

Methods:

It would be useful to clarify in the methods section that in the Serratia model the bacteria will not infect if the PM is compromised.

*We added a sentence with references and indication that the detailed description of how Serratia model works is provided in the discussion section.*

p. 22 line 406 State the full name of the EP gene.

*EP is the full name. It represents the tandem repeats of glutamic acid-proline (EP) dipeptides.*

p. 29 Line 552 It would be useful for the reader to include some specific examples of similar models where bacteria could be genetically modified.

*We included references in now line 547.*

p. 33 Line 659 With the current data, it is not proven that the antagomir is constitutively produced, only one time point was evaluated.

*The constitutive reduction is implied because recSodalis is continuously replicating and secreting molecules into the fly. We added a clarification “recSodalis continuously produces miR275 sponges” into the text.*

References: all species should be in italics, and some references have a different format (ex. line 885). The paratransgenic approach using densovirus (Lie et al) is a relevant reference to include.

*We have added this reference (31) as requested by the reviewer.*

Line 994 Correct student´s to Student´s t test

*We have made this change as requested by the reviewer.*

Figure 7 "gut tissue specific" should be reworded, as other tissues were not tested. It might be better to state that the system does not affect expression in salivary glands at the tested timepoint. Effects at other time points or tissues were not tested.

*We changed the 3.7 subtitle and Figure 7 to “Paratransgenic knockdown of miR275 is not observed in tsetse’s salivary glands”. We also rephrased lines 527-529 (now lines 521-523) accordingly.*

There are several paragraphs in the results section that should be in the discussion section. For ex.

p. 26 line 490 "Higher levels..."

p. 25 line 465 "All of these..."

p. 24 line 461 "Morover..."

p. 23 Line 435 "These up and downregulated..."

*We have made these changes as requested by the reviewer.*

Figure 1. cfu/midgut does not normalize for the size of the tissues. For the reader to better appreciate the differences in bacterial loads between the two tissues, it would be better to normalize using tissue mg as denominator.

*We agree with the reviewer that it would be a good idea to normalize by tissue weight. However, tsetse’s cardia organ is too small to accurately weigh. Our method reflects total bacterial load per organ, whereas the reviewer’s suggested method would reflect relative bacterial load per mg of organ. Although the later method may normalize the bacterial load in the two organs, it would not change the fact that the midgut houses a higher absolute number of bacteria than does the cardia, which means more antagomir-275 in the midgut (compared to cardia) to knockdown miR275.*

Figure 1 D and E. Is it possible that no difference was observed in expression in the cardia because the tissue amount was too low? The legend indicates that 5 cardia and 5 midguts were used, but these tissues most probably have different sizes that could affect the efficiency of the qPCR method if it is not normalized by mg of tissue. p. 12 Line 210 "A small portion of the RNA" may not allow for comparison across tissues. This should be addressed as a possible limitation or potentially include previous optimization results for the qPCR to show that this variability in input RNA does not affect the efficiency of the qPCR.

*We specified “the small portion of RNA” by modifying the sentence to “100 ng and 500 ng of cardia and midgut RNA, respectively” (now P12 Line 217). We think the qPCR quantification of miR275 is sufficient in our study. We did not make a direct comparison of miR275 expression levels between cardia and midgut, thus we feel that it is not necessary to normalize by mg of tissue. The comparison of miR275 abundance between two tissues was made in our previous publication (as cited reference 10 in the discussion section).*
